# Multi-Fish Tracking in Complex Underwater Environments Using BF-DETR and DAPS-Enhanced BoT-SORT

**DOI:** 10.3390/ani16213347

**Published:** 2026-10-23

**Authors:** Binhong Zhou, Xiang Liu, Yuling Liu, Yihan Yan, Jiaxian Mu, Xiaoyan Chen

**Affiliations:** 1 College of Information Engineering, Sichuan Agricultural University, Ya’an 625014, China; 202406649@stu.sicau.edu.cn (B.Z.); 202406804@stu.sicau.edu.cn (X.L.); 202406837@stu.sicau.edu.cn (Y.L.); 202502131@stu.sicau.edu.cn (J.M.); 2 College of Food Science, Sichuan Agricultural University, Ya’an 625014, China; 202501630@stu.sicau.edu.cn

**Keywords:** fish, multi-object tracking, fish re-identification, underwater object detection, tracking-by-detection, fish behavior analysis, intelligent aquaculture

## Abstract

Reliable multi-object tracking of fish is essential for individual-level behavior analysis and intelligent aquaculture monitoring. However, degraded underwater imagery, ambiguous boundaries in dense groups, and high inter-individual appearance similarity often lead to missed detections, association errors, and identity switches. We propose BF-DETR-DAPS-BoT-SORT, a tracking-by-detection framework integrating fish detection, identity representation, and trajectory association. BF-DETR incorporates a Fish Morphology Enhancement Module (FMEM) to model local texture, directional structure, and contextual information, and a Boundary-Contrast Separation Module (BCSM) to distinguish adjacent individuals. We further develop the Detail-Aware Part Similarity Network (DAPS), which combines underwater texture enhancement, dual-axis local representations, and part-similarity learning to capture subtle appearance differences under changes in swimming direction. DAPS features are integrated into BoT-SORT for data association. On task-specific datasets constructed from continuous underwater videos, BF-DETR achieved mAP@0.5 and mAP@0.5:0.95 values of 96.63% and 77.53%, respectively. The complete framework achieved MOTA, HOTA, and IDF1 scores of 93.1%, 75.8%, and 91.2%, respectively, with 13 identity switches. Compared with BoT-SORT, HOTA and IDF1 increased by 1.4 and 2.1 percentage points, while identity switches decreased from 18 to 13. These results indicate improved trajectory continuity and identity consistency in complex underwater scenes, supporting reliable individual-level fish behavior analysis.

## Simple Summary

Monitoring how individual fish move can help researchers study behavior and improve aquaculture management. However, tracking fish in underwater videos is difficult because individuals often look similar, overlap, and become partly hidden. Reflections and unclear images make this task even harder. We developed a method that combines improved fish detection with recognition of subtle visual differences between individuals. Tests on videos recorded in laboratory tanks showed that the method improved fish detection and maintained more consistent individual identities across successive frames. Compared with the existing tracking method, it reduced the number of times that fish identities were incorrectly switched from 18 to 13. More continuous and reliable movement records could support future studies of swimming activity, group behavior, and responses to feeding. These potential applications require further validation, including testing whether improved tracking leads to more accurate behavioral measurements and whether the method works reliably in commercial aquaculture environments.

## 1. Introduction

As aquaculture becomes increasingly large-scale, intensive, and intelligent, continuous non-invasive monitoring of farmed fish using computer vision has become an important area of research. Individual trajectories reflect activity states, spatial distributions, and behavioral changes. They also provide essential data for feeding analysis, growth assessment, health monitoring, and abnormal behavior recognition [1,2,3]. Compared with manual observation, video-based monitoring supports long-term data acquisition while causing less disturbance to natural fish activity. It is therefore better suited to automated monitoring in group-rearing environments. Accurate localization of multiple fish and consistent identity assignment across video frames are prerequisites for individual-level behavioral analysis.

Recent advances in deep learning and computer vision have promoted the application of multi-object tracking (MOT) to underwater fish monitoring. Most fish MOT methods follow a tracking-by-detection paradigm. They first localize fish and then combine motion and appearance cues for cross-frame identity matching. Li et al. proposed TFMFT, which uses a Transformer architecture to improve multi-fish association in complex scenes [3]. Liu et al. proposed FishTrack, which jointly models motion, spatiotemporal, and appearance information to improve tracking under occlusion and body deformation [4]. Huang et al. introduced the Uniform and Deformable Multi-Fish Tracking (UD-MFT) benchmark to evaluate real-time tracking under appearance similarity, body deformation, occlusion, and disappearance [5]. More recently, MFT25 introduced a dedicated benchmark and tracking framework for nonlinear motion, occlusion, and appearance similarity in underwater fish [6]. These studies have advanced fish MOT. However, underwater imaging conditions and fish morphology differ markedly from those encountered in terrestrial tracking. Reliable detection and identity consistency under challenging conditions therefore remain major obstacles to long-term tracking.

Complex underwater environments pose substantial challenges for fish detection. Light attenuation, glass reflections, background variation, and occlusion can degrade texture and edge information. Fish are also elongated, non-rigid targets with pronounced pose variation, and dense groups frequently involve contact, crossing, and partial overlap. Previous studies have improved underwater fish detection through feature enhancement, multiscale fusion, and attention mechanisms [7,8,9]. Li et al. incorporated RT-DETR feature modeling to improve detection under occlusion [7]. Another study by Li et al. used prior-knowledge enhancement to address blur and occlusion in high-density aquaculture [8]. Ruan et al. used deformable convolutions to improve robustness to body deformation [9]. Nevertheless, enhancing whole-object features alone cannot fully resolve feature confusion where neighboring fish meet. Elongated non-rigid fish require both stronger internal morphological representations and clearer boundary discrimination during close contact or partial overlap. Missed detections, localization errors, and merged targets can also propagate to downstream data association.

In addition to detection quality, high appearance similarity among conspecific fish hinders cross-frame identity consistency. Individuals often share similar body shapes and colors, leaving fine local textures and subtle markings as the primary identity cues. These cues are sensitive to reflections, low contrast, occlusion, and viewpoint changes. Fan et al. introduced the FS48 fish re-identification (ReID) dataset and FSNet, which integrates multiview information to improve individual recognition. They also showed that environmental and pose changes increase the difficulty of fish ReID [10]. In person and vehicle ReID, PCB improves fine-grained identity representation through part-level feature learning [11]. Such studies demonstrate the value of local-region modeling for fine-grained recognition. Unlike people or vehicles, however, fish lack prominent identity markers, and swimming-direction changes alter local-region correspondence. Stable association therefore requires detailed local representations that remain robust to pose variation.

Detection and identity representation ultimately affect data association in MOT. Wu et al. combined detection and ReID cues in an improved DeepSORT framework for dynamic fish identification and counting [12]. Liu et al. optimized the DeepSORT ReID network to reduce identity confusion caused by appearance similarity and occlusion in high-density aquaculture [13]. Xing et al. combined an improved YOLOv8 detector with BoT-SORT for fish-school detection, tracking, and counting in complex sonar environments [14]. These studies show that fish MOT depends on both the association strategy and the quality of detection and appearance features. In dense underwater groups, detection errors reduce the reliability of spatial observations. Weak appearance discrimination also causes incorrect associations and identity switches after crossing or occlusion. Improving detection and individual representation together is therefore essential for stable long-term fish tracking.

To address these challenges, we propose a multi-object tracking framework for complex multi-fish underwater scenes that improves cross-frame association through detection and appearance representation. First, we develop the BF-DETR fish detection network. Its Fish Morphology Enhancement Module (FMEM) and Boundary-Contrast Separation Module (BCSM) strengthen fish morphology representation and boundary discrimination between neighboring targets. Second, we develop the Detail-Aware Part Similarity Network (DAPS) for individual fish re-identification. Detail-aware enhancement, dual-axis local representations, and part-similarity learning improve appearance discrimination and robustness to changes in swimming direction. Finally, DAPS appearance features and BF-DETR detections are incorporated into BoT-SORT [15]. The resulting framework integrates underwater fish detection, individual representation, and trajectory association.

The main contributions of this study are as follows:

(1) We construct a task-specific dataset suite for underwater fish multi-object tracking. Continuously recorded multi-fish videos are divided by sequence to create separate detection and MOT datasets. Track-level identity annotations are further used to construct a fish ReID dataset, enabling independent evaluation of each task.
(2) We propose the BF-DETR fish detection network. FMEM and BCSM address weak morphological information and boundary confusion between neighboring fish by strengthening structural representation and boundary separation in complex underwater scenes.
(3) We propose the DAPS network for individual fish re-identification and integrate it into the fish MOT framework. DAPS strengthens fine-grained identity features and local discriminative information, improving differentiation between similar individuals and supporting more stable cross-frame association with BoT-SORT.

## 2. Materials and Methods

### 2.1. Data Acquisition

Continuous videos of swimming fish groups were recorded in an indoor laboratory tank setting in Ya’an, Sichuan Province, China, in September 2025. The dataset included multiple fish species collectively known locally as “Jiluo”, including *Neolissochilus stracheyi*, *N. baoshanensis*, *N. heterostomus*, *N. qiaojiensis*, *Tor sinensis*, *T. douronensis*, *T. yingjiangensis*, and *Spinibarbus denticulatus*. Fish lengths ranged approximately from 5 to 50 cm, and estimated ages ranged from approximately 2 months to 3 years. Rectangular glass tanks of several sizes were used to record multi-fish scenes at different spatial scales. Representative tank dimensions (length × width × height) were 0.60 × 0.35 × 0.35 m, 1.20 × 0.50 × 0.50 m, and 2.05 × 0.81 × 0.90 m.

Videos were captured using Sony α6400 (Sony Corporation, Tokyo, Japan) and Canon EOS M6 Mark II (Canon Inc., Tokyo, Japan) digital cameras. Each camera was mounted on a tripod approximately 1 m from the tank and positioned either directly in front of the tank or at an oblique frontal angle. Fish activity was recorded from a horizontal viewpoint at a resolution of 1920 × 1080 pixels and a frame rate of 30 frames/s.

**Figure 1:**
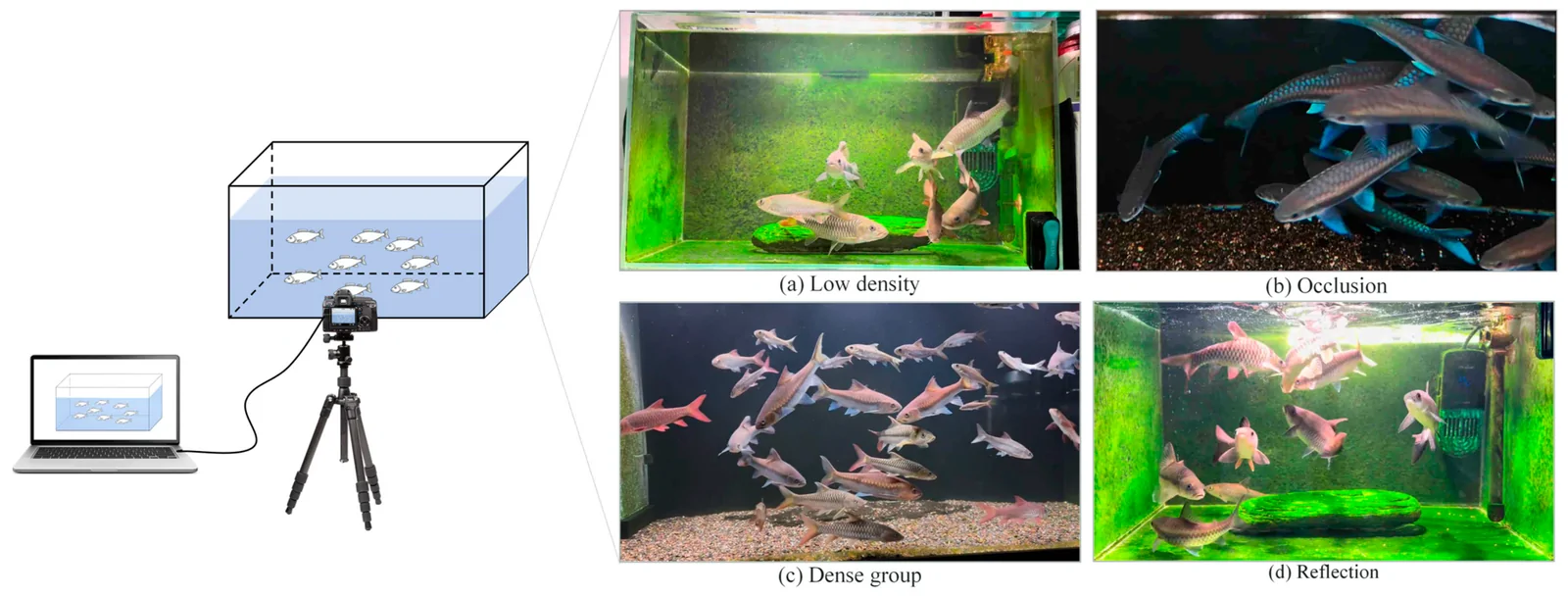
Data acquisition setup and representative underwater fish scenes. (**a**) Low-density scene; (**b**) occlusion scene; (**c**) dense-group scene; (**d**) reflection scene.

The recorded scenes varied in fish density, background structure, ambient illumination, and water clarity. The videos included fish crossing, partial and severe occlusion, dense aggregation, complex backgrounds, and reflections from the tank walls and water surface. Figure 1 shows the recording setup and representative scenes. Figure 1a–d present low-density, occlusion, dense-group, and reflection scenarios, respectively. During video acquisition, the fish were allowed to swim freely without external stimulation or behavioral manipulation.

### 2.2. Dataset Construction

The continuous multi-fish videos were used to construct datasets for detection, multi-object tracking, and individual re-identification. The detection dataset and the MOT test set did not share any original recordings. For both datasets, all recordings from a given acquisition session were assigned to a single subset. Physical tanks were shared across subsets, but acquisition sessions were kept separate. Track-level identity annotations from the MOT data were then used to crop individual fish images and construct the DAPS ReID dataset.

The detection dataset was constructed from 20 source recordings, with 12 assigned to training, four to validation, and four to testing. All frames sampled from a given recording were retained within a single subset. Detection images were sampled from the detection sequences at intervals of 50 frames. Fish bounding boxes were annotated using LabelImg (version 1.8.1). Sampling yielded 1372 training images, 171 validation images, and 172 test images, totaling 1715 images and corresponding to an approximate image-count ratio of 8:1:1.

Offline augmentation was applied only to the training set to improve robustness to illumination changes, motion blur, target scale, and viewpoint variation. Blur, color, and geometric transformations produced 1670 augmented samples. Together with the original images, they formed a training set of 3042 samples. Table 1 summarizes the dataset split and augmented sample counts.

The MOT dataset was constructed from continuous video sequences and annotated frame by frame using DarkLabel (version 2.4). Each fish was assigned a bounding box and a track ID that was maintained while its identity could be reliably established. Crossings, severe occlusions, and reappearances were examined using preceding and subsequent frames, considering motion continuity and distinguishable appearance cues. When identity continuity remained uncertain after review, the original track was terminated and a new track ID was assigned. A new track ID was not taken as evidence of a different physical individual. The 12 independently recorded videos were partitioned as complete sequences, with four recordings assigned to each of the training, validation, and test sets. Each recording was retained entirely within a single subset, as summarized in Table 2. Video durations range from 10 to 66 s, with 7–15 annotated track IDs per sequence. The scenes include gravel and submerged decorations, green backgrounds, and bright or purple illumination, together with fish overlap, occlusion, and reflections. Track IDs were recoded by combining the video-sequence number with the original ID to ensure uniqueness across sequences. During manual review, annotations were checked for missing or inaccurate bounding boxes, identity inconsistencies, and duplicate use of the same track ID for different fish within a frame. Any identified errors were corrected. Particular attention was paid to crossings, severe occlusions, and reappearances.

Finally, fish regions were cropped from consecutive frames using the annotated track identities in the MOT training sequences. ReID crops were retained only when the individual identity was unambiguous and sufficient appearance information was visible. Crops with uncertain identities, severe blur, strong glare obscuring identifying features, severe occlusion or truncation, or overlap that prevented identification of the target individual were excluded. Partially occluded samples and samples with pose variation were retained when identity and appearance cues remained reliable. The ReID training and test sets contained distinct physical fish, with no individual overlap. The training set contained 29 identities, while the test set contained 13 unseen identities and was further divided into query and gallery sets. For each test trajectory, the first 60% was used to construct the gallery set, and the final 20% was used to construct the query set. The middle 20% was excluded from both sets as a temporal buffer. Table 3 provides the detailed statistics.

### 2.3. Overall Framework of the Proposed Method

Video MOT methods can broadly be categorized as tracking-by-detection (TBD) or joint detection and tracking. TBD methods detect targets in each frame and combine appearance and motion cues for cross-frame association. This design can handle occlusion, temporary target loss, and complex motion. Frequent directional changes, crossing, occlusion, and appearance similarity make TBD particularly suitable for long-term underwater fish monitoring. The paradigm jointly exploits detection results and identity representations during association [1,3,4,6,16].

**Figure 2:**
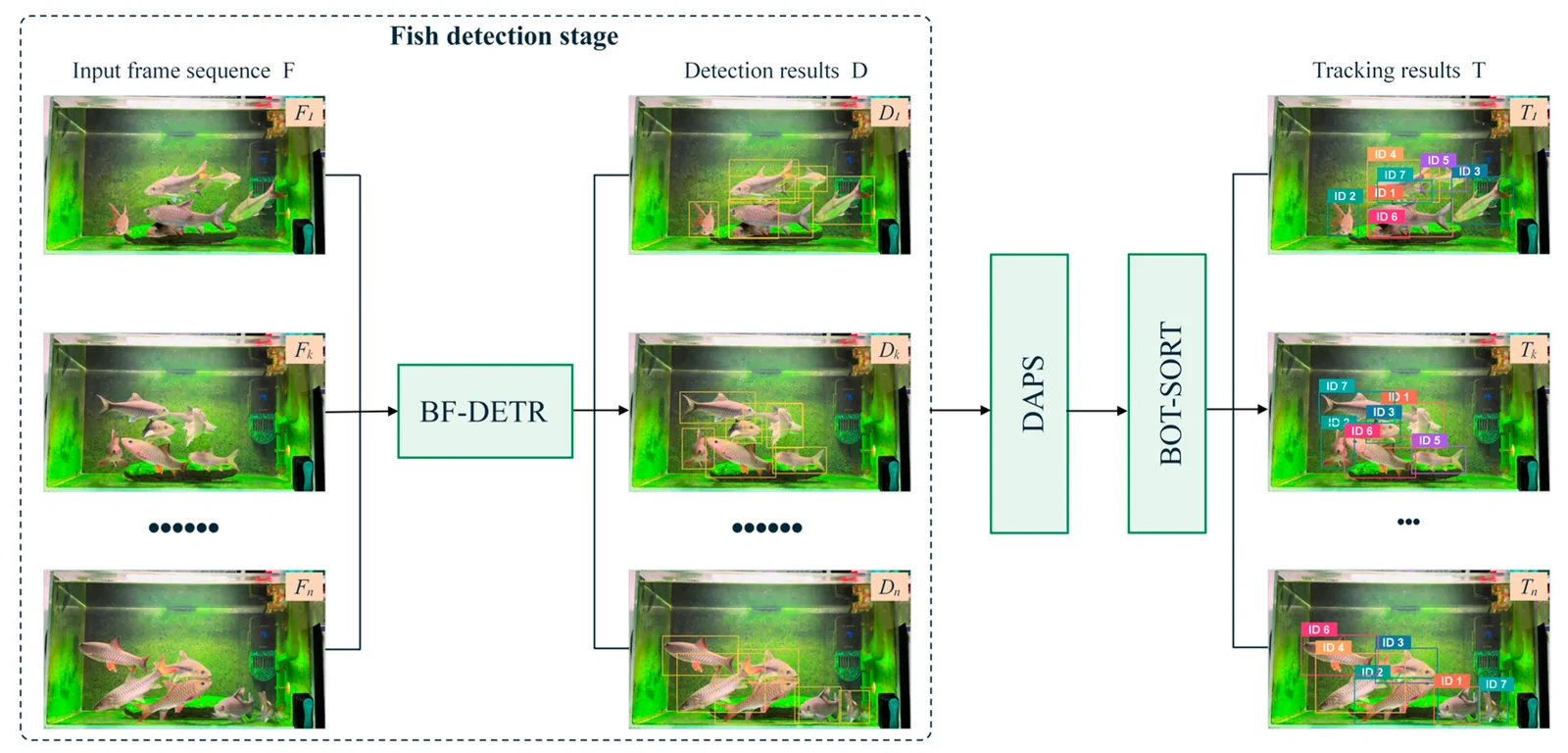
Framework of the proposed underwater fish multi-object tracking method.

We propose a TBD-based framework for underwater fish multi-object tracking with three components: fish detection, individual re-identification, and multi-object association. BF-DETR detects fish in each frame and outputs their locations and confidence scores. DAPS extracts appearance features to distinguish visually similar individuals. BoT-SORT combines detections, appearance features, and motion information to associate targets across frames and generate stable trajectories. Figure 2 presents the overall framework.

### 2.4. BF-DETR Fish Detection Network

#### 2.4.1. Overall Architecture of BF-DETR

**Figure 3:**
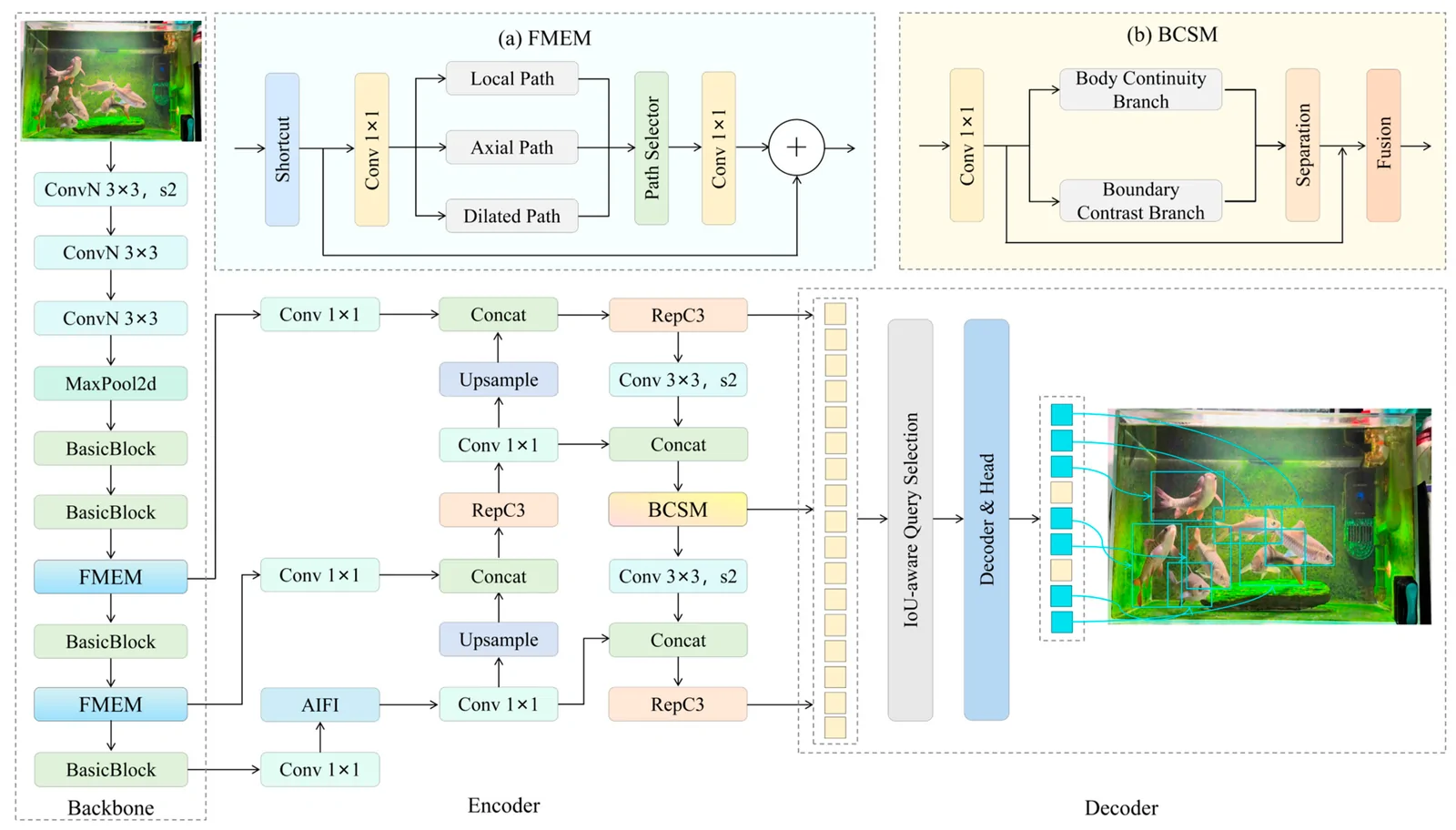
Overall architecture of the proposed BF-DETR network.

We construct BF-DETR using RT-DETR-R18 as the baseline detector to address degraded morphological cues and boundary ambiguity between neighboring fish. BF-DETR retains the end-to-end RT-DETR-R18 architecture, comprising a backbone, a hybrid encoder, and a Transformer decoder [17]. FMEM is inserted after the P3/8 and P4/16 backbone outputs. It integrates local texture, directional structure, and contextual information to strengthen fish morphology at shallow and intermediate feature scales. BCSM is introduced during P4 feature fusion in the CCFF component of the hybrid encoder. It models contrast between boundary regions and their neighborhoods while suppressing feature confusion where adjacent fish meet. The enhanced multiscale features are then encoded and decoded for end-to-end classification and bounding-box regression. Figure 3 shows the complete BF-DETR architecture.

#### 2.4.2. Fish Morphology Enhancement Module (FMEM)

Underwater fish are typically elongated and non-rigid, with pronounced pose variation. Their detection depends on local texture and edges as well as complete internal structural representations. In complex underwater scenes, light attenuation, reflections, background interference, and occlusion weaken local feature responses. The resulting missing or discontinuous internal information reduces the detector’s ability to perceive overall fish morphology [7,8,9].

Conventional convolutions extract local spatial features using fixed-scale receptive fields. Although effective for textures and edges, they inadequately model relationships among the head, trunk, and tail of elongated fish. Simply enlarging the receptive field introduces context but can also increase interference from neighboring targets and complex backgrounds. Underwater fish detection therefore requires feature enhancement that jointly models local details, directional structure, and contextual information.

**Figure 4:**
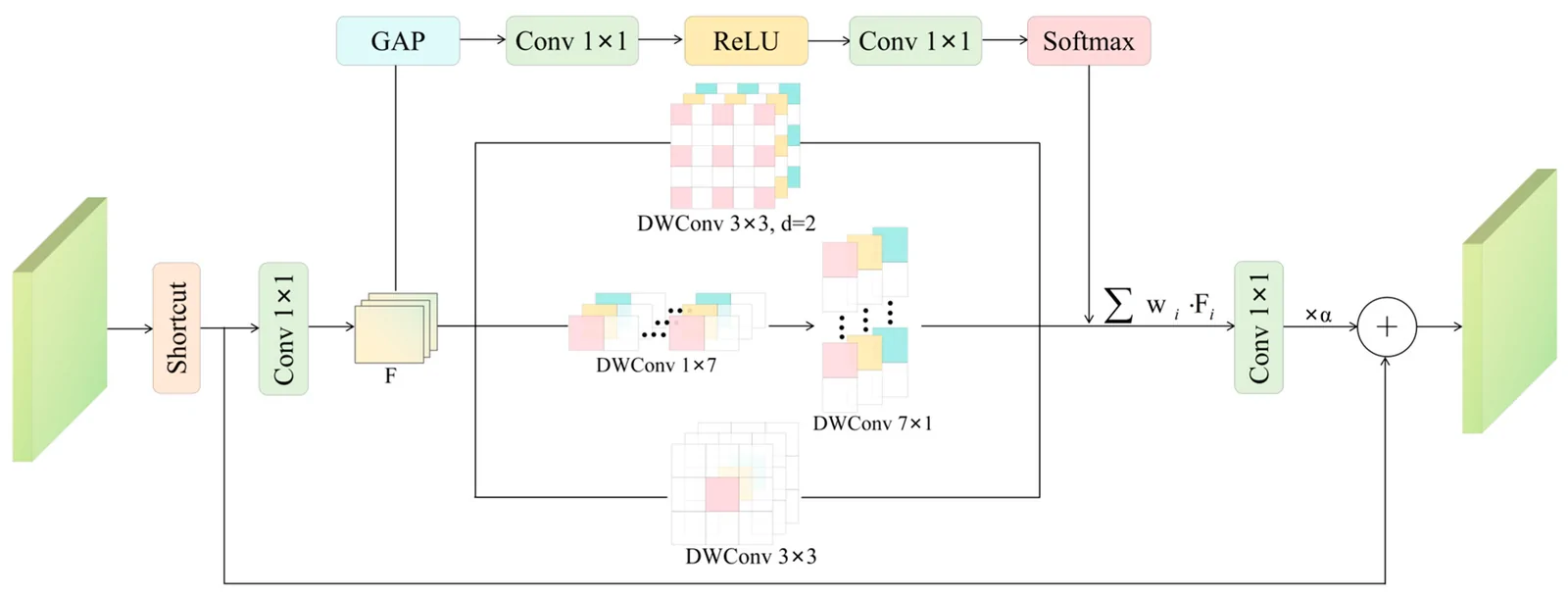
Overall architecture of the Fish Morphology Enhancement Module (FMEM).

We therefore propose the FMEM. As shown in Figure 4, FMEM uses a multibranch architecture to enhance local texture, directional structure, and contextual information. Their relative importance varies with pose, swimming state, and imaging conditions. FMEM consequently uses MorphologySelector to assign dynamic branch weights and select the most informative morphological representation for each input.

Given an input feature X, FMEM first uses a 1×1 convolution for channel compression. Three parallel morphology branches are then applied to the compact feature. The Local Path uses depthwise convolution to extract local textures and edge details. The Axial Path uses asymmetric convolutions to model longitudinal structure and spatial relationships among the head, trunk, and tail. The Dilated Path uses dilated depthwise convolution to enlarge the receptive field and obtain context at low computational cost. Together, the three branches preserve local details and overall fish structure.

Because the importance of morphological cues varies across underwater scenes, MorphologySelector adaptively weights the three branches. It compresses spatial information through global average pooling and generates branch responses with two 1×1 convolutions. Softmax then produces a dynamic weight for each branch:

(1) $$w=Softmax(Conv_{1\times 1}(ReLU(Conv_{1\times 1}(GAP(F)))))$$

where $w=\{w_{l},w_{a},w_{d}\}$ are the selection weights for the Local, Axial, and Dilated Paths, respectively. The three branches are adaptively fused as follows:

(2) $$F_{m}=w_{l}F_{l}+w_{a}F_{a}+w_{d}F_{d}$$

where $F_{l}$, $F_{a}$, and $F_{d}$ denote the local-texture, directional-structure, and contextual features extracted by the three branches, respectively.

Finally, a 1×1 convolution restores the channel dimension, and the fused morphological feature is combined with the original feature through a residual connection:

(3) $$Y=X+\alpha Conv_{1\times 1}(F_{m})$$

where α is a learnable scaling parameter that controls the contribution of the enhanced feature relative to the original semantic information.

FMEM strengthens underwater fish representations through local texture, directional structure, and contextual relationships. Its dynamic morphology selection adapts the contribution of each cue to pose and imaging conditions. This design improves structural perception of elongated non-rigid fish and provides more reliable features for localization and boundary separation in dense groups.

#### 2.4.3. Boundary-Contrast Separation Module (BCSM)

FMEM strengthens the structural representation of individual fish through multidirectional morphology modeling. However, substantial feature confusion can remain between neighboring individuals in dense groups. When fish are close or partially occluded, their feature responses interfere during fusion and produce similar boundary representations. This interference reduces localization and discrimination of individual targets. Enhancing only within-object structure is therefore insufficient to separate neighboring fish in complex underwater scenes [7,8,9].

We propose the BCSM to improve feature separability between neighboring fish in dense groups. Unlike feature extractors that focus only on appearance enhancement, BCSM combines structural-continuity modeling with boundary-contrast enhancement. It jointly optimizes within-target consistency and between-target differences. Directional fish structure maintains a complete representation of each target, while differences between local semantics and neighboring responses strengthen discriminative features at contact boundaries.

**Figure 5:**
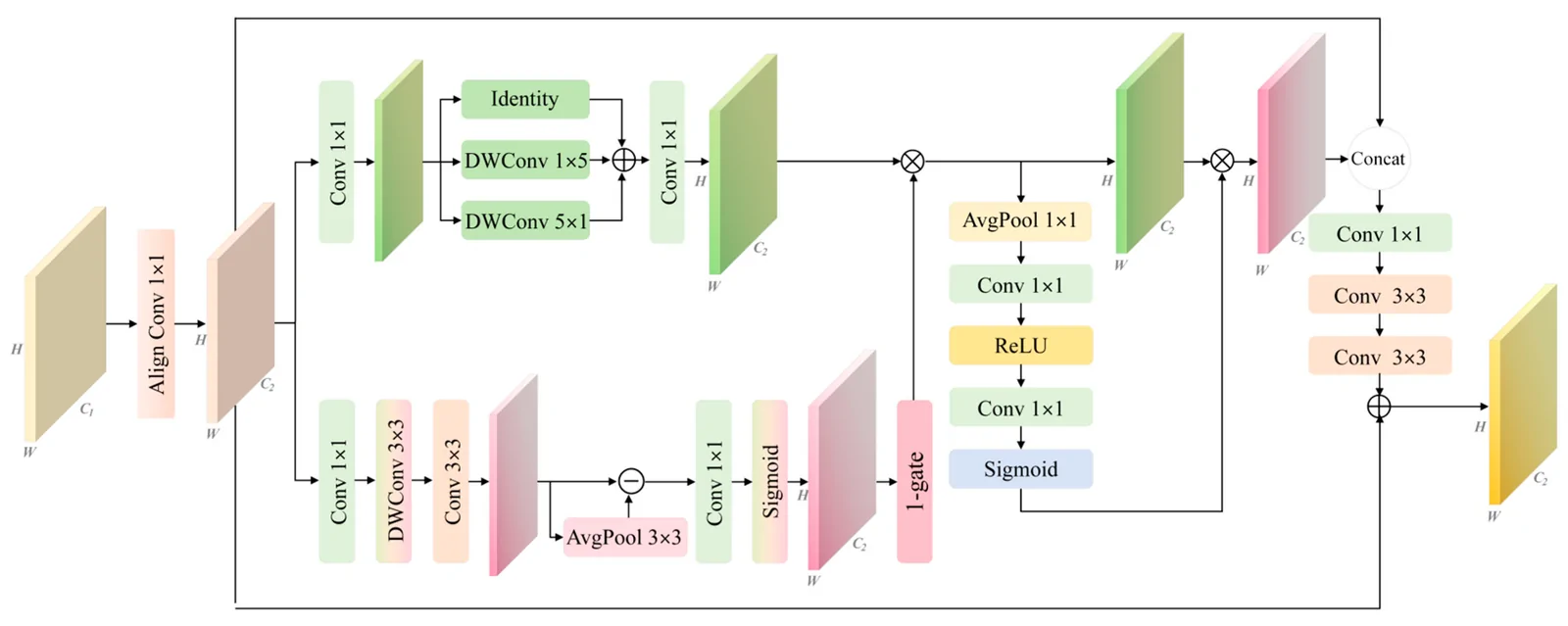
Overall structure of the Boundary-Contrast Separation Module (BCSM).

As shown in Figure 5, BCSM first adjusts the channels of the input feature and then performs feature separation through structural-continuity modeling, boundary-contrast enhancement, and adaptive feature modulation. Given $F_{in}$, a 1×1 convolution maps the channels to obtain the base feature:

(4) $$F_{a}=Conv_{1\times 1}(F_{in})$$

where $F_{a}$ denotes the channel-adjusted base feature.

The head, trunk, and tail of an elongated fish exhibit clear spatial continuity, which standard convolutions capture inadequately. BCSM therefore uses a structural-continuity path. An identity branch preserves the original response, while asymmetric strip depthwise convolutions extract spatial continuity along different directions. The 1×5 and 5×1 convolutions capture directional structural dependencies as follows:

(5) $$F_{c}=Conv_{1\times 1}(F_{id}+DWConv_{1\times 5}(F_{u})+DWConv_{5\times 1}(F_{u}))$$

where $F_{u}$ is the adjusted input, $F_{id}$ is the identity-mapping branch, and $F_{c}$ is the structural-continuity feature. This path preserves the main fish representation while strengthening internal structural consistency, providing a stable constraint for feature separation.

BCSM further introduces a boundary-contrast mechanism to differentiate neighboring fish. A convolution first extracts local semantic features, and local average pooling then obtains neighborhood context. Their difference is calculated as follows:

(6) $$F_{d}=F_{l}-AveragePool(F_{l})$$

where $F_{d}$ denotes the boundary-contrast feature. Unlike gradient-based edge extraction, this operation models response differences between target and surrounding regions directly in deep semantic space. It therefore captures discriminative boundaries under low contrast, occlusion, and target contact.

After obtaining structural-continuity and boundary-contrast features, BCSM generates gating weights from the boundary response to modulate the structural feature adaptively. The gate suppresses the spread of structural information at contact boundaries, reducing confusion between individuals. Within fish regions, it retains more continuity information to preserve a complete target representation.

A lightweight channel-recalibration mechanism then refines the effective responses. Feature fusion and a residual connection produce the final output without excessively altering the original target representation. This process improves the module’s adaptation to complex underwater conditions.

By combining structural-continuity modeling, semantic boundary contrast, and adaptive modulation, BCSM preserves fish structure while improving discrimination between neighboring individuals. It alleviates blurred boundaries and feature confusion in dense groups, providing more accurate and stable detections for downstream multi-object tracking.

### 2.5. Detail-Aware Part Similarity Network (DAPS)

In multi-object tracking, the ReID module learns appearance features for identity association across video frames. In underwater fish groups, high inter-individual similarity, pose variation, occlusion, and changing image quality make association based only on location and motion prone to identity confusion [10,12,13]. Stable discriminative features are therefore essential for trajectory continuity. We develop DAPS to address subtle appearance differences, weak local textures, and unstable part correspondence under pose changes. DAPS combines detail enhancement, global modeling, dual-axis local representations, and part-similarity constraints. The resulting appearance features are supplied to BoT-SORT for data association.

**Figure 6:**
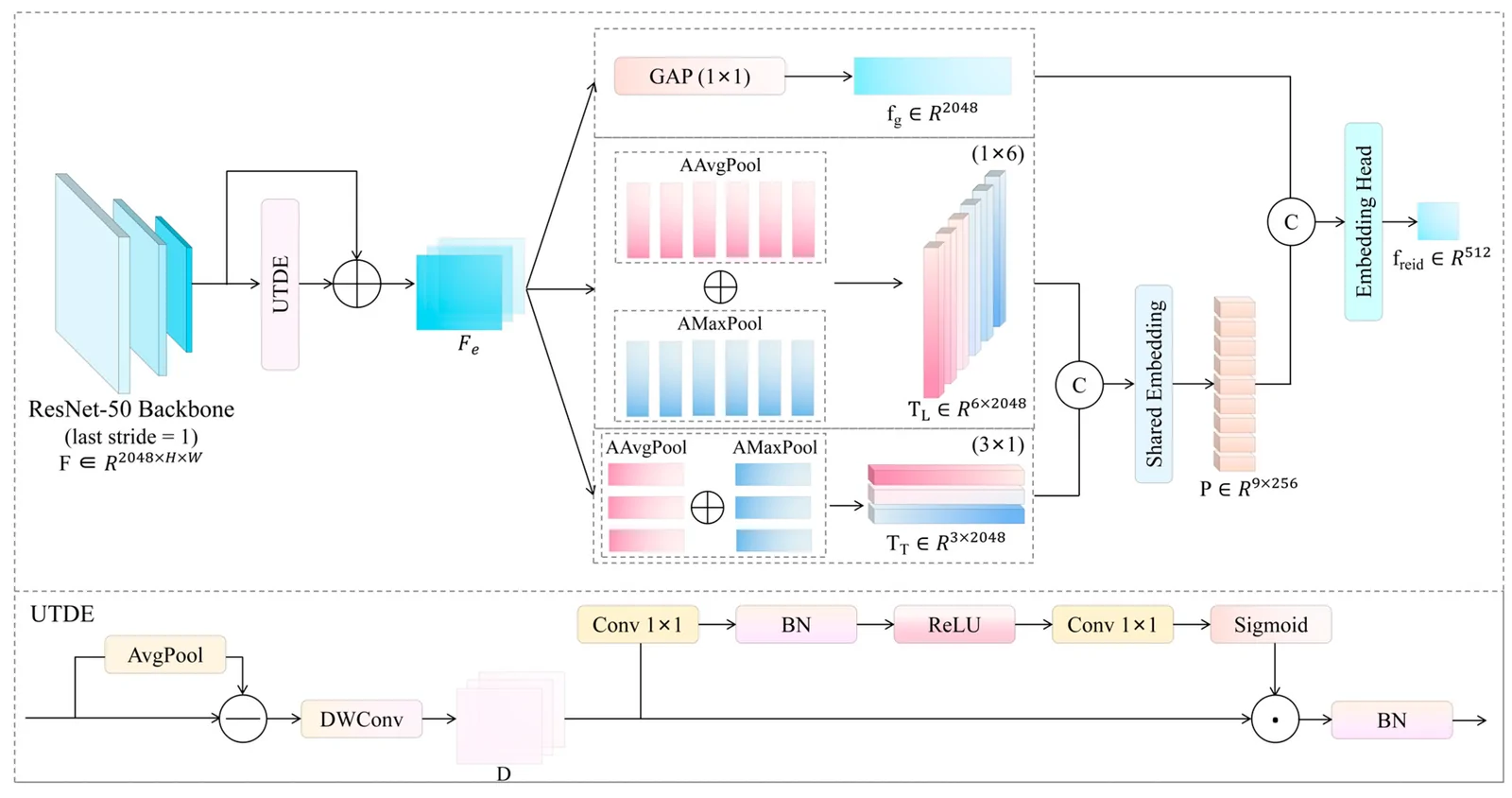
Overall structure of the Detail-Aware Part Similarity Network (DAPS).

Figure 6 shows the DAPS architecture. A ResNet-50 backbone [18] extracts high-level semantic features. The stride of its final stage is set to 1 to retain spatial detail for fish ReID. The Underwater Texture Detail Enhancement module (UTDE) then enhances local texture cues weakened by light attenuation, scattering, and reflections. DAPS subsequently constructs a global feature branch and dual-axis local branches to model overall appearance and local structure. Shared embedding projections and feature fusion produce the final identity representation.

DAPS combines a global feature with local features obtained by pooling along two fixed image-coordinate axes. The longitudinal branch produces six tokens along the feature-map width, and the transverse branch produces three tokens along its height. The six-region longitudinal and three-region transverse partitions were selected as a heuristic design to provide finer spatial subdivision along the elongated fish body while retaining broader spatial context across its shorter dimension. When the fish body is approximately aligned with the image width, the longitudinal sequence can roughly reflect the spatial progression from head to tail. These pooled regions provide approximate spatial partitions rather than explicitly segmented anatomical parts. The local tokens pass through a shared embedding layer and are fused with the global feature. During training, forward and reversed correspondence are compared only for the six longitudinal tokens to accommodate opposite head-tail ordering; this does not provide invariance to arbitrary rotation or explicitly compensate for body curvature. However, the model does not explicitly estimate regional visibility or exclude occluded regions. Substantial rotation, pronounced curvature, or severe occlusion may therefore weaken anatomical correspondence.

Changes in swimming direction can reverse the arrangement of local regions for the same fish across frames. DAPS therefore uses part-similarity learning to constrain local correspondence. Local features are first normalized, and cosine similarity measures corresponding parts between two samples. For Equations (7)–(9), let $P_{i}$ and $P_{j}$ denote the sequences of six longitudinal feature tokens from samples *i* and *j*, respectively (K=6). Reversed correspondence reverses only this six-token sequence. The three transverse tokens contribute to local identity classification and feature fusion but are excluded from the part-alignment loss. The forward part similarity is defined as follows:

(7) $$S_{f}(i,j)=\frac{1}{K}\sum_{k=1}^{K}\;\;cos(p_{i}^{k},p_{j}^{k})$$

where $p_{i}^{k}$ and $p_{j}^{k}$ denote the features of the kth local region in the two samples. To account for directional changes, DAPS also matches the local-region sequence in reverse order:

(8) $$S_{r}(i,j)=\frac{1}{K}\sum_{k=1}^{K}\;\;cos(p_{i}^{K+1-k},p_{j}^{k})$$

where $S_{r}(i,j)$ denotes local-region correspondence under reversed ordering. DAPS selects the larger of the forward and reverse similarities and converts it into a distance:

(9) $$d_{ij}=1-max(S_{f}(i,j),S_{r}(i,j))$$

The distance defined in Equation (9) is used to construct a batch-hard part-alignment loss. For each valid anchor, the loss selects the most distant positive sample and the nearest negative sample within the batch:

(10) $$L_{\mathrm{align}}=\frac{1}{\left|V\right|}\sum_{i\in V}{\left[\underset{j\in P_{i}}{max}\;d_{ij}-\underset{n\in N_{i}}{min}\;d_{in}+m\right]}_{+},m=0.3$$

Here, $P_{i}$ contains samples with the same identity as anchor i, excluding the anchor itself, and $N_{i}$ contains samples with different identities. The set V includes anchors having both positive and negative samples in the batch, and ${\left[x\right]}_{+}=max(0,x)$. The alignment loss is set to zero if no valid anchors are available.

Local-region supervision is applied to all nine embedded tokens using independent identity classifiers. The local-region loss is the mean identity classification cross-entropy over the batch and the nine regions:

(11) $$L_{\mathrm{part}}=-\frac{1}{9B}\sum_{i=1}^{B}\sum_{k=1}^{9}log\;q_{i,k}(y_{i})$$

Here, B is the batch size, $y_{i}$ is the identity label of sample *i*, and $q_{i,k}(y_{i})$ is the softmax probability assigned to the true identity by the classifier for region *k*.

Finally, DAPS fuses the global and local features, and an embedding head generates the identity representation. Network parameters are optimized using the following joint loss:

(12) $$L=L_{id}+L_{tri}+0.20L_{part}+0.05L_{align}$$

where $L_{id}$, $L_{tri}$, $L_{part}$, and $L_{align}$ denote the identity classification, triplet, local-region supervision, and part-alignment losses, respectively. During inference, DAPS supplies appearance embeddings to BoT-SORT to maintain identity consistency across consecutive frames. The part distance in Equation (9) is used for training supervision, whereas inference-time association uses the fused appearance embeddings.

### 2.6. DAPS-Enhanced BoT-SORT Fish Multi-Object Tracking

DAPS appearance embeddings are incorporated into BoT-SORT data association [15] to form the complete fish MOT framework. For each frame, BF-DETR first outputs fish locations and confidence scores. DAPS then extracts an appearance feature from each detected region. BoT-SORT combines these detections and features with motion states to match targets across frames. For existing tracks, a Kalman filter predicts the current locations, and matched detections update their states. Figure 2 presents the overall tracking process.

During the first association stage, BoT-SORT matches high-confidence detections. For the ith existing track and the jth current detection, the cosine distance between their DAPS appearance features is calculated as follows:

(13) $$d_{app}(i,j)=1-\frac{f_{i}^{T}f_{j}}{\left||f_{i}||||f_{j}|\right|}$$

where $f_{i}$ and $f_{j}$ are the DAPS feature vectors of the existing track and current detection, respectively. A smaller $d_{app}$ indicates greater similarity in feature space. BoT-SORT then combines appearance similarity, Kalman-predicted locations, and spatial constraints between boxes to identify candidate matches. Each detection can therefore be associated with an existing track using both motion and appearance cues.

After the first stage, unmatched tracks are compared with low-confidence detections. Such detections often arise from occlusion, missing local regions, or reduced detection quality, making their appearance features less stable. This stage consequently relies mainly on spatial overlap between predicted tracks and detection boxes. Staged association of high- and low-confidence detections [19] exploits useful observations while limiting the effect of erroneous matches on trajectory continuity.

After association, matched tracks update their motion states and appearance representations using the current detections. Unmatched detections initialize candidate tracks, whereas tracks that remain unmatched are retained or removed according to the management rules. BF-DETR thus provides reliable observations, DAPS supplies discriminative identity representations, and BoT-SORT fuses appearance and motion for cross-frame association. Together, they form a complete pipeline from detection and individual representation to trajectory association.

## 3. Results and Analysis

### 3.1. Experimental Environment and Parameter Settings

All experiments were conducted on a Linux workstation with an NVIDIA GeForce RTX 4090 GPU (NVIDIA Corporation, Santa Clara, CA, USA). The software environment comprised Python 3.10.14, PyTorch 2.2.2, CUDA 11.8, and cuDNN 8.5. BF-DETR used an input size of 640×640, a batch size of 16, and 200 training epochs. AdamW was used with an initial learning rate of $1\times 10^{-4}$. DAPS was trained for 200 epochs with a batch size of 96 using SGD and an initial learning rate of 0.001.

### 3.2. Evaluation Metrics

The BF-DETR-DAPS-BoT-SORT framework was evaluated at the detection, individual re-identification, and multi-object tracking levels.

For detection, Precision (*P*), Recall (*R*), F1-score, mAP@0.5, and mAP@0.5:0.95 [20] were used to evaluate BF-DETR. The number of parameters measured model size. Precision, Recall, and F1-score were calculated as follows:

(14) $$P=\frac{TP}{TP+FP}$$

(15) $$R=\frac{TP}{TP+FN}$$

(16) $$F1=\frac{2PR}{P+R}$$

where TP, FP, and FN denote the numbers of correct detections, false detections, and missed targets, respectively.

Average Precision (AP) was calculated from the precision–recall curve. Mean Average Precision (mAP) is the mean AP across all classes and was calculated as follows:

(17) $$mAP=\frac{1}{C}\sum_{i=1}^{C}\;\;AP_{i}$$

where *C* is the number of classes and $AP_{i}$ is the average precision for class *i*. mAP@0.5 denotes mAP at an IoU threshold of 0.5. mAP@0.5:0.95 averages AP over IoU thresholds from 0.5 to 0.95 in increments of 0.05, providing a stricter assessment of localization. The parameter count reflects model size.

For individual re-identification, DAPS was evaluated using mAP and Rank-k accuracy derived from the cumulative matching characteristic curve. ReID mAP measures the overall retrieval ranking of each query within the gallery. Rank-k is the proportion of queries whose correct identity appears among the top k results. We report Rank-1, Rank-5, and Rank-10 [21].

For multi-object tracking, overall performance was evaluated using Higher Order Tracking Accuracy (HOTA), Multiple Object Tracking Accuracy (MOTA), Identification F1 score (IDF1), and identity switches (IDSW).

MOTA accounts for false negatives, false positives, and identity switches and was calculated as follows [22]:

(18) $$MOTA=1-\frac{FN+FP+IDSW}{GT}$$

where FN, FP, IDSW, and GT denote false negatives, false positives, identity switches, and ground-truth targets, respectively.

HOTA jointly evaluates detection accuracy (DetA) and association accuracy (AssA) and was calculated as follows [23]:

(19) $$HOTA=\sqrt{DetA\times AssA}$$

where DetA and AssA denote detection and association accuracy, respectively.

IDF1 measures trajectory identity preservation and was calculated as follows [24]:

(20) $$IDF1=\frac{2IDTP}{2IDTP+IDFP+IDFN}$$

where IDTP, IDFP, and IDFN are identity-level true positives, false positives, and false negatives, respectively. IDSW denotes the number of identity switches during tracking. Higher MOTA, HOTA, and IDF1 indicate better performance, whereas fewer identity switches indicate stronger identity consistency.

### 3.3. BF-DETR Detection Results

#### 3.3.1. Performance Comparison with Different Detection Models

BF-DETR was compared with representative detectors to evaluate underwater fish detection. The baselines included the two-stage Faster R-CNN [25]; YOLOv9m, YOLOv10m, YOLOv11m, and YOLOv12m [26,27,28,29]; D-FINE-M [30]; and RT-DETR-R18, RT-DETR-R34, and RT-DETR-L [17]. All models used identical data splits, training strategies, and experimental environments to ensure a fair comparison. Table 4 reports the results.

Table 4 shows clear performance differences among the detectors. Faster R-CNN produced relatively low accuracy in complex underwater scenes, with mAP@0.5 and mAP@0.5:0.95 values of 87.23% and 49.92%. Both the YOLO and Transformer-based models performed better. YOLOv11m achieved an mAP@0.5 of 95.54%, while RT-DETR-R34 and RT-DETR-L reached 95.88% and 95.69%, respectively.

Underwater fish are elongated non-rigid targets with pronounced pose variation and ambiguous boundaries between neighbors. General-purpose detectors may therefore fail to capture fish morphology fully or separate adjacent individuals in dense scenes. Based on RT-DETR-R18, BF-DETR uses FMEM to strengthen morphological representations and BCSM to improve boundary discrimination.

BF-DETR achieved the best overall detection performance. Precision, Recall, mAP@0.5, mAP@0.5:0.95, and F1-score reached 95.17%, 93.40%, 96.63%, 77.53%, and 94.28%, respectively, with 21.15 M parameters. Compared with RT-DETR-R18, BF-DETR added only 1.25 M parameters, approximately 6.3%. It improved mAP@0.5 from 94.90% to 96.63%, mAP@0.5:0.95 from 72.90% to 77.53%, and Recall from 90.76% to 93.40%. These gains support the effectiveness of enhanced structural and boundary representations.

Compared with the larger RT-DETR-L, BF-DETR used approximately 34.1% fewer parameters and improved mAP@0.5:0.95 by 2.40 percentage points. These results suggest that the gain arose from fish-specific feature enhancement rather than network scaling alone. Because detection quality directly affects association and trajectory preservation, BF-DETR also provides more reliable input for DAPS and BoT-SORT.

#### 3.3.2. Grad-CAM-Based Feature Activation Visualization of Different Detection Models

**Figure 7:**
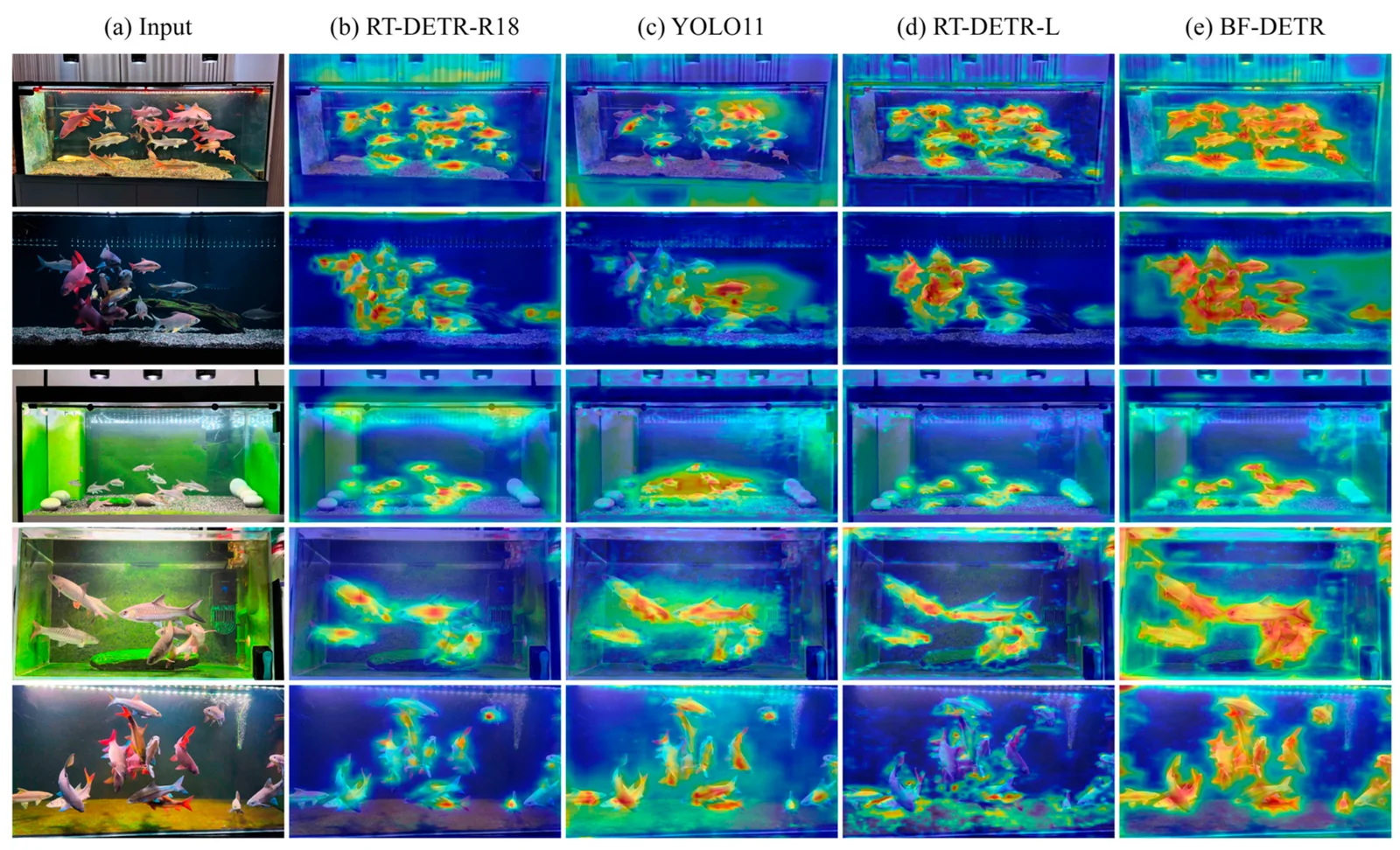
Grad-CAM visualization of feature activation maps for different detection models. (**a**) Input images; (**b**) RT-DETR-R18; (**c**) YOLO11; (**d**) RT-DETR-L; (**e**) BF-DETR.

To examine feature learning across detectors, Grad-CAM [31] was used to visualize responses from YOLO11, RT-DETR-R18, RT-DETR-L, and BF-DETR. Figure 7 presents the results. Warm colors indicate stronger responses and reveal the regions emphasized by each detector.

Figure 7 shows distinct response patterns among the detectors. RT-DETR-R18 and YOLO11 responded to fish regions, but their activations were less complete in dense groups, low-contrast scenes, and complex backgrounds. Some fish structures lacked continuous high-response regions. The larger RT-DETR-L produced richer responses in certain scenes, although its activations sometimes spread beyond the target.

BF-DETR produced more stable target activation across underwater conditions. In Figure 7e, high-response regions covered the main fish structure more completely and remained focused in dense and low-contrast scenes. These patterns indicate that BF-DETR learned more effective fish representations.

Overall, BF-DETR generated more complete and concentrated responses than the comparison models, indicating more discriminative fish representations. This visualization is consistent with the quantitative detection results.

#### 3.3.3. Ablation Study of FMEM and BCSM

Ablation experiments were conducted using RT-DETR-R18 as the baseline to quantify the contributions of FMEM and BCSM. Each module and their combination were evaluated under identical experimental conditions. Table 5 reports the results.

FMEM targets insufficient morphological representation, whereas BCSM addresses boundary confusion in dense scenes. The single-module and combined settings therefore reveal their independent and complementary effects.

RT-DETR-R18 achieved an mAP@0.5 of 94.90% and an mAP@0.5:0.95 of 72.90%. Adding FMEM increased these metrics to 95.67% and 75.16%, respectively. The 2.26-percentage-point gain in mAP@0.5:0.95 indicates better representation of fish structure under pose and appearance variation.

Adding BCSM increased mAP@0.5 and mAP@0.5:0.95 to 96.15% and 76.16%, respectively. The 3.26-percentage-point improvement in mAP@0.5:0.95 under stricter IoU thresholds indicates better boundary discrimination between neighboring fish in dense groups.

Combining FMEM and BCSM produced the best overall performance. Relative to RT-DETR-R18, Recall increased from 90.76% to 93.40%, F1-score from 92.20% to 94.28%, mAP@0.5 by 1.73 percentage points, and mAP@0.5:0.95 by 4.63 percentage points. The final mAP values were 96.63% and 77.53%. Precision was slightly lower than with BCSM alone, but Recall, F1-score, and both mAP metrics improved. FMEM therefore helped balance detection completeness and localization accuracy.

FMEM and BCSM address complementary limitations in underwater fish detection. FMEM strengthens morphological structure, whereas BCSM separates ambiguous target boundaries. Their combination improves both structural representation and discrimination in dense scenes, providing more reliable detections for DAPS feature extraction and BoT-SORT association.

#### 3.3.4. Visualization of FMEM and BCSM Contributions

**Figure 8:**
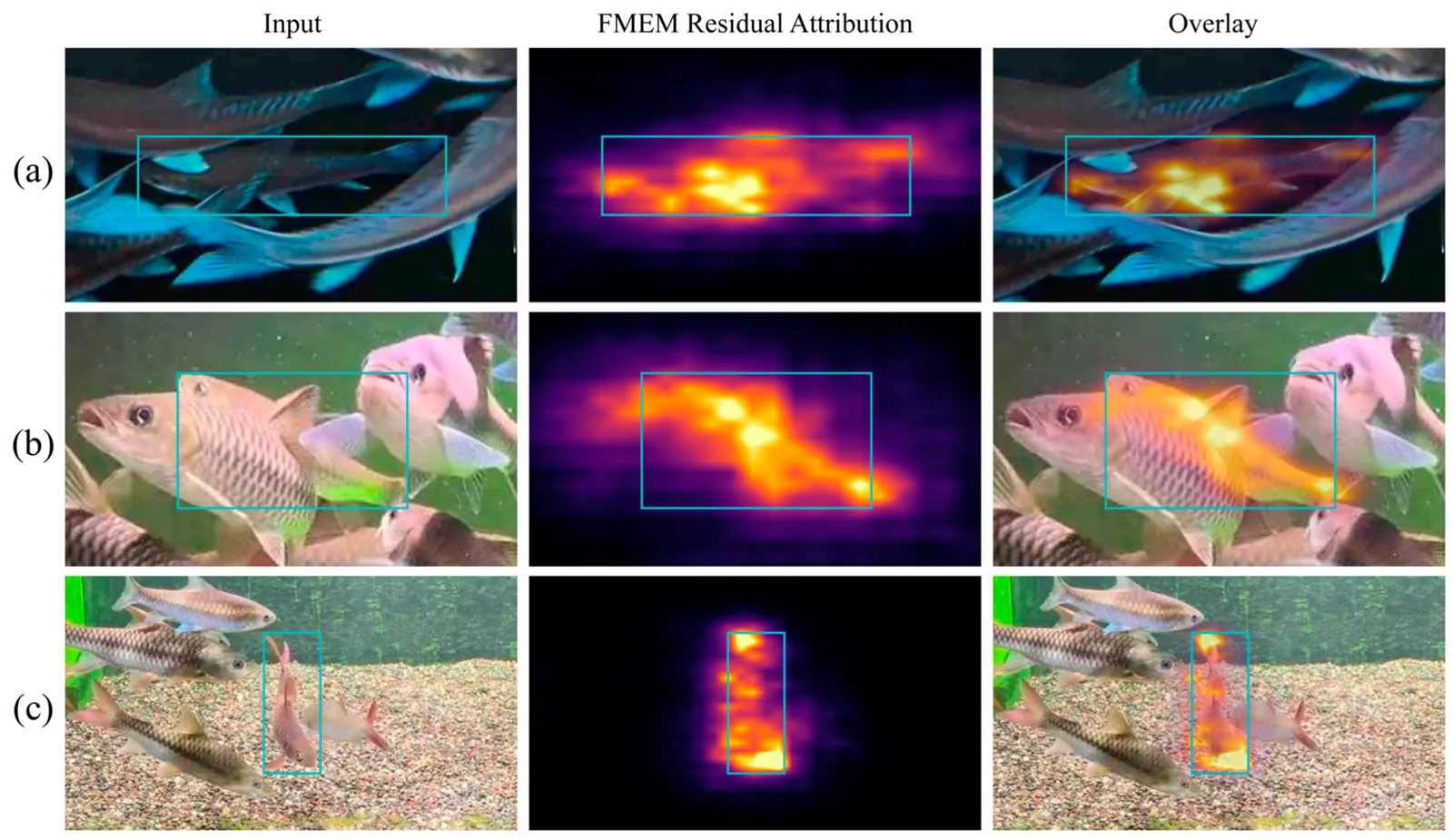
Visualization of FMEM residual attribution. (**a**) A fish in a near-horizontal orientation; (**b**) a fish in an oblique orientation; (**c**) a fish in a near-vertical orientation.

**Figure 9:**
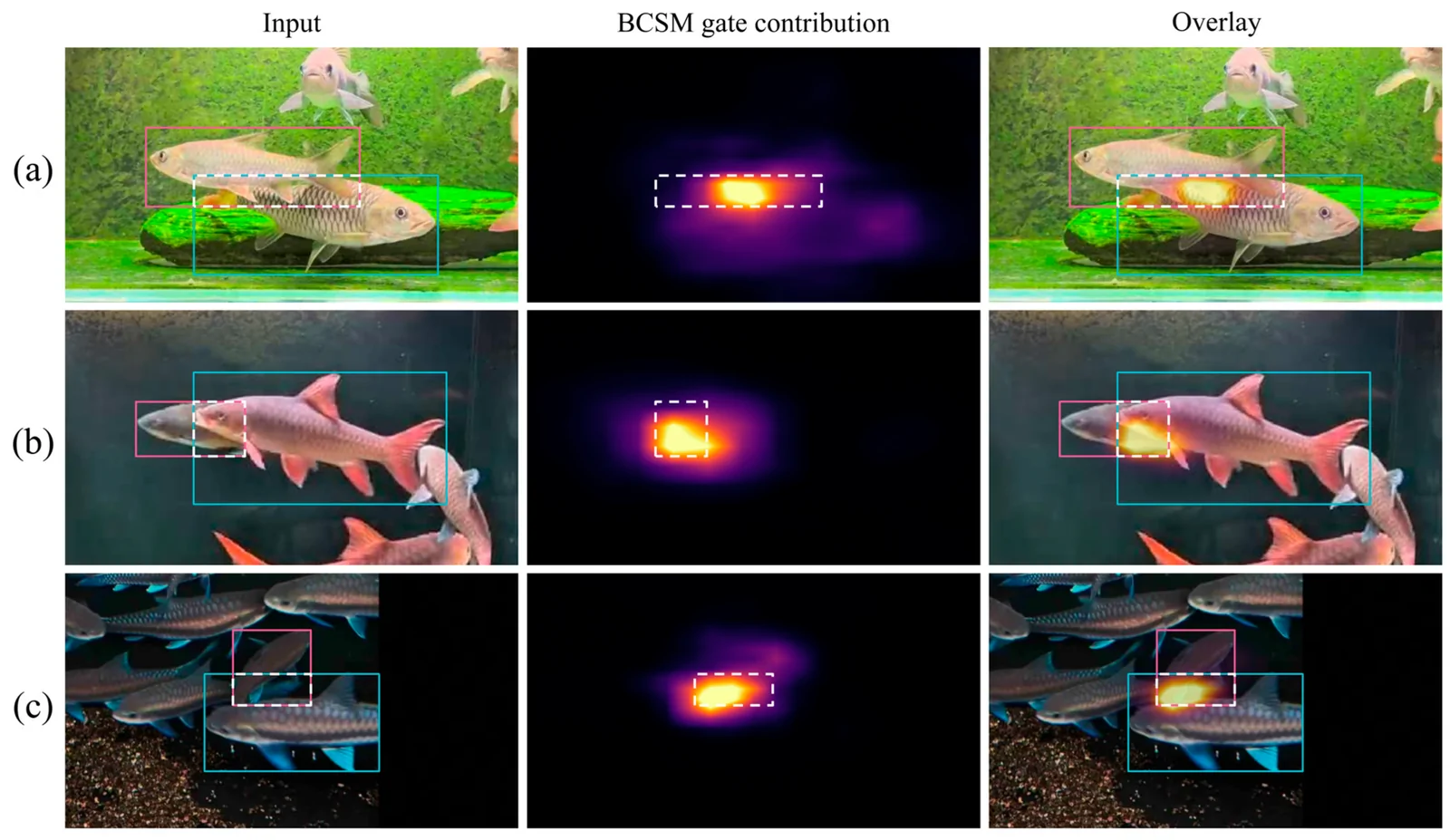
Visualization of BCSM gate contribution. (**a**) Overlap between fish body regions; (**b**) overlap near fish heads; (**c**) overlap among fish in a dense group.

To examine how FMEM and BCSM contribute to BF-DETR, we visualized the feature responses generated by each module. Their response regions reveal how the modules affect fish feature extraction. Figure 8 and Figure 9 present the FMEM and BCSM visualizations, respectively.

As shown in Figure 8, FMEM residual-attribution responses were concentrated on fish regions. Across different poses and scales, the module responded strongly to body contours, trunks, and local morphological regions. Its responses remained aligned with fish structure under partial occlusion and pose changes. FMEM therefore strengthened the representation of internal structure and appearance cues used for detection.

As shown in Figure 9, BCSM gating responses were concentrated on target boundaries and interactions between neighboring fish. When fish overlapped or were closely spaced, strong responses occurred primarily at ambiguous contact regions rather than across the entire target. BCSM therefore emphasized boundary-related differences and improved separation of neighboring individuals in dense groups.

FMEM and BCSM enhanced BF-DETR in complementary ways. FMEM captured fish morphology and improved the completeness of target representations, whereas BCSM emphasized inter-target boundaries and increased separability between neighboring individuals. The visualizations illustrate their distinct contributions to underwater fish detection.

#### 3.3.5. Qualitative Analysis of Detection Results Under Challenging Scenarios

**Figure 10:**
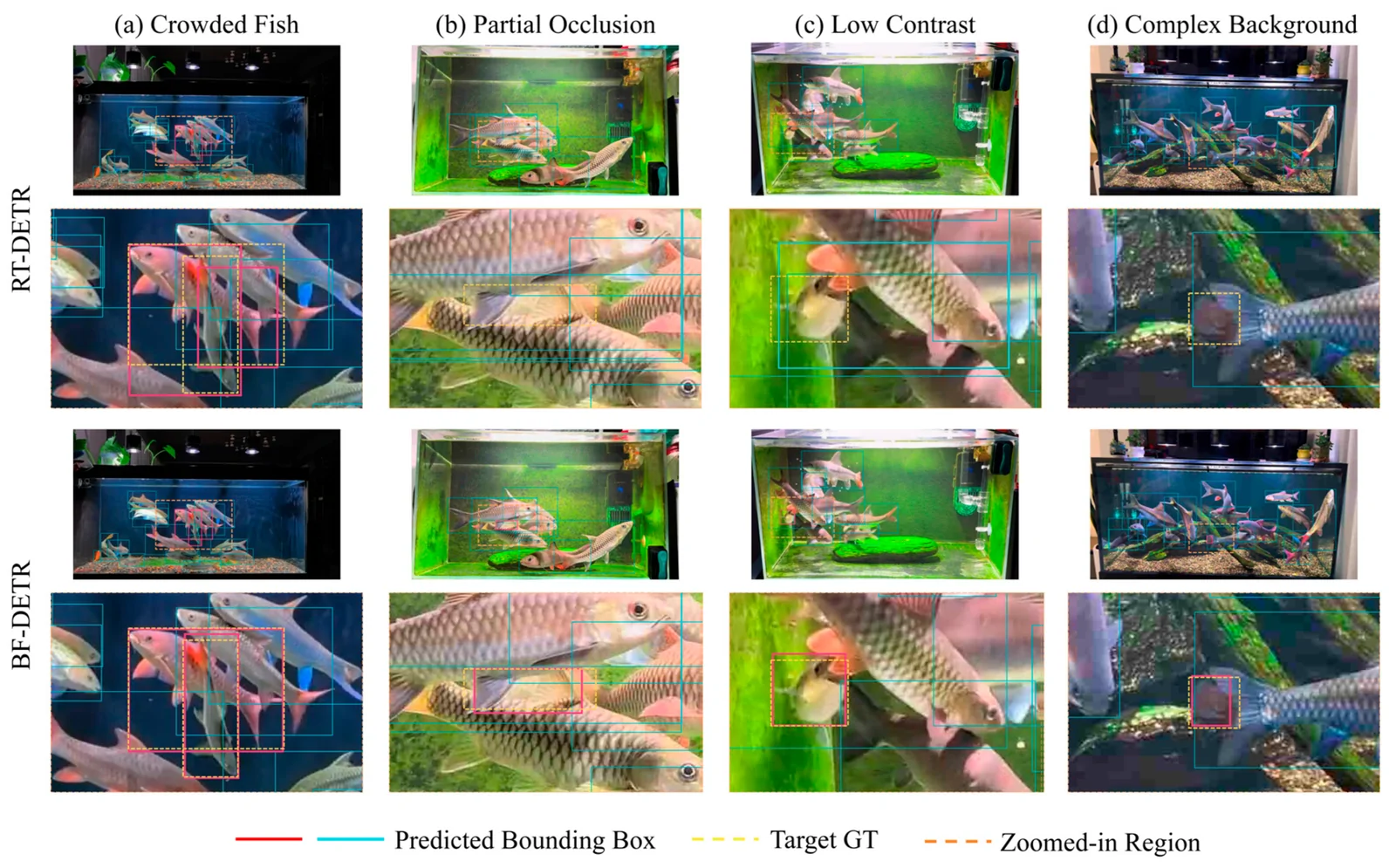
Qualitative comparison of detection results between RT-DETR and BF-DETR under challenging underwater scenarios. (**a**) Crowded fish; (**b**) partial occlusion; (**c**) low contrast; (**d**) complex background.

BF-DETR was qualitatively evaluated in four representative challenging scenarios: dense groups, partial occlusion, low contrast, and complex backgrounds. Figure 10 compares RT-DETR and BF-DETR. Cyan and red boxes indicate predictions, yellow dashed boxes indicate selected ground-truth boxes, and orange dashed boxes mark enlarged regions.

In the dense-group scene (Figure 10a), substantial overlap caused boundary ambiguity between neighboring fish. RT-DETR produced several inaccurate bounding boxes that did not fully cover the corresponding individuals. In contrast, BF-DETR separated neighboring fish more clearly and achieved more accurate localization.

In the partial-occlusion scene (Figure 10b), interference from surrounding fish reduced the visible area of the partially occluded individual. RT-DETR failed to detect this fish, whereas BF-DETR retained the detection, suggesting greater robustness when only limited visual features remained visible.

In the low-contrast scene (Figure 10c), weak visual differences between the fish and its surroundings obscured the target boundary. RT-DETR missed a weakly visible fish, whereas BF-DETR retained the detection, demonstrating greater robustness to low-contrast conditions in this example.

In the complex-background scene (Figure 10d), aquatic plants, decorations, and water textures introduced substantial visual interference. RT-DETR missed one of the fish, whereas BF-DETR detected it successfully, indicating greater robustness to background interference.

Overall, BF-DETR produced more stable detections in dense, occluded, low-contrast, and complex-background scenes. It reduced missed detections and improved target localization across the evaluated challenging conditions.

### 3.4. DAPS Re-Identification Results

#### 3.4.1. Performance Comparison with Different Re-Identification Models

DAPS was compared with ResNet-50 [18], PCB [11], AlignedReIDNet [32], and MGN [33] for individual fish re-identification. These methods represent baseline convolutional feature extraction, local-part modeling, feature alignment, and multigranularity learning, respectively. Performance was evaluated using mAP, Rank-1, Rank-5, and Rank-10. Table 6 reports the results.

ResNet-50 learned useful appearance features but remained sensitive to illumination changes, pose variation, and inter-individual similarity. PCB, AlignedReIDNet, and MGN improved performance through local-region modeling, feature alignment, and multigranularity fusion, respectively. DAPS achieved the highest mAP, indicating more discriminative fish identity representations.

Rank-based performance also differed across the ReID models. Methods using local features or multigranularity representations outperformed ResNet-50, showing that a single global feature inadequately captures subtle individual differences. DAPS achieved the best Rank-1 accuracy and maintained strong Rank-5 and Rank-10 performance, indicating accurate and stable identity retrieval.

Among the evaluated methods, DAPS achieved the best overall performance for individual fish re-identification. Its fish-specific fine-grained learning enhances local appearance information and integrates global and local representations. These results support DAPS as a reliable appearance-feature extractor for subsequent BoT-SORT data association.

#### 3.4.2. Ablation Study of DAPS Components

To examine the contributions of individual DAPS components, we compared five configurations: the global baseline, DAPS without UTDE, DAPS with only the longitudinal branch, DAPS with forward-only part correspondence, and the complete DAPS. The longitudinal-only configuration removes the transverse branch while retaining longitudinal part alignment. The forward-only configuration retains both local branches and the part-alignment loss, disabling only reversed correspondence. Performance was evaluated using mAP and Rank-1 on the overall ReID test set.

As shown in Table 7, the complete DAPS achieved an mAP of 71.76% and a Rank-1 accuracy of 85.45%. Removing UTDE reduced these metrics by 2.58 and 3.44 percentage points, respectively. Retaining only the longitudinal branch reduced mAP and Rank-1 by 1.89 and 2.65 percentage points, indicating the benefit of incorporating the complementary transverse representation. Using forward-only correspondence reduced the two metrics by 1.74 and 2.91 percentage points. These comparisons support the contributions of UTDE, dual-axis representation, and reverse part matching to overall ReID performance.

### 3.5. Fish Multi-Object Tracking Results

#### 3.5.1. Comparison with Different Multi-Object Tracking Algorithms

The proposed method was compared with ByteTrack [19], OC-SORT [34], StrongSORT [35], BoT-SORT [15], DeepSORT [36], and BoostTrack [37] for fish multi-object tracking. For a fair comparison, all tracking methods in Table 8 were evaluated using identical frame-level bounding boxes and confidence scores generated by BF-DETR. MOTA, HOTA, IDF1, and IDSW were used as evaluation metrics. Table 8 reports the results.

The methods differed modestly in MOTA but more clearly in HOTA, IDF1, and IDSW, which reflect association and identity consistency. DeepSORT and StrongSORT achieved IDF1 values of 72.2% and 77.5% and produced 106 and 84 identity switches, respectively. These results show the difficulty of maintaining identity when similar fish approach, cross, or occlude one another. ByteTrack, OC-SORT, and BoostTrack achieved high MOTA but lower HOTA and IDF1 than our method. High overall tracking accuracy therefore did not necessarily correspond to stable association.

Among the baselines, BoT-SORT produced the strongest overall results, with MOTA, HOTA, and IDF1 values of 93.0%, 74.4%, and 89.1% and 18 identity switches. Our method increased MOTA to 93.1%, a gain of only 0.1 percentage points. In contrast, HOTA and IDF1 increased by 1.4 and 2.1 percentage points to 75.8% and 91.2%, respectively. The number of identity switches decreased by approximately 27.8%, from 18 to 13. The main benefit over BoT-SORT was therefore improved association quality and identity consistency rather than a large MOTA gain.

Across all metrics, the proposed method maintained high overall tracking accuracy while providing clearer gains in association and identity preservation. Higher HOTA and IDF1 together with fewer identity switches indicate more stable cross-frame identity consistency.

#### 3.5.2. Qualitative Comparison of Different Multi-Object Tracking Algorithms

**Figure 11:**
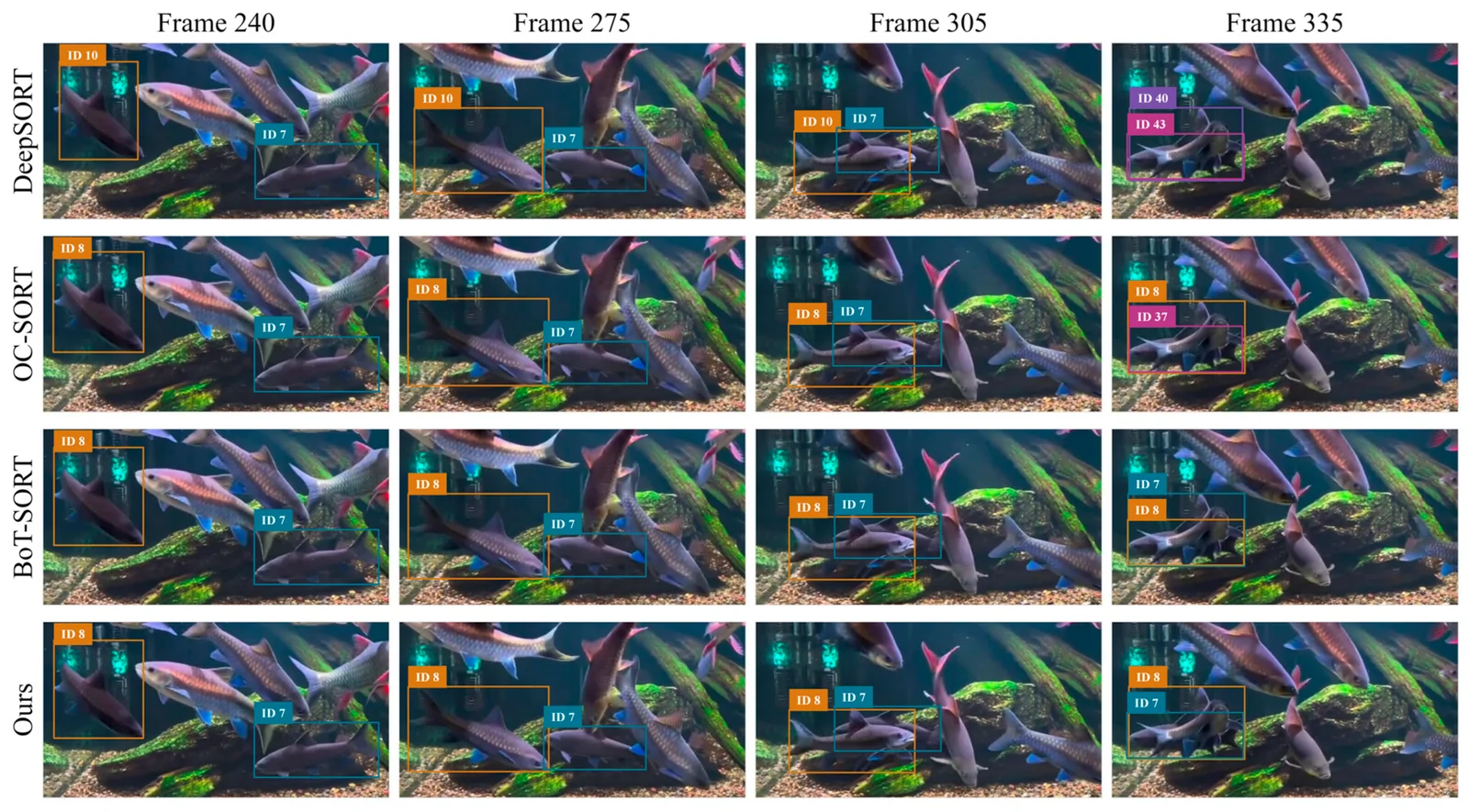
Qualitative comparison of tracking results of different multi-object tracking algorithms on consecutive underwater fish frames.

A continuous underwater video sequence was used to compare DeepSORT, OC-SORT, BoT-SORT, and the proposed method qualitatively. Figure 11 presents changes in target IDs across consecutive frames, revealing each method’s ability to preserve identity during fish interactions.

Figure 11 shows fish approaching and interacting while swimming, creating identity ambiguity between similar individuals. DeepSORT exhibited clear identity changes. The targets retained IDs 10 and 7 from Frames 240 to 305 but changed to IDs 40 and 43 in Frame 335. DeepSORT was therefore susceptible to identity switches in complex multi-fish scenes.

OC-SORT used motion information to improve trajectory prediction and maintained more continuous tracks than DeepSORT. It retained IDs 8 and 7 in the initial frames but switched identities in later frames. Motion consistency alone was therefore insufficient to associate visually similar fish reliably.

BoT-SORT combined appearance and motion cues and provided better stability in this sequence. However, the two IDs were exchanged in Frame 335 during target interaction. Appearance features did not completely prevent identity confusion under fish similarity and pose variation.

The proposed method maintained more stable identity assignments throughout the sequence. The two targets consistently retained IDs 8 and 7 in Frames 240, 275, 305, and 335, with no visible exchange. DAPS therefore learned more discriminative appearance features and improved identity distinction between similar fish.

#### 3.5.3. Ablation Study of BF-DETR and DAPS in the Tracking Framework

Four BoT-SORT-based settings were evaluated to isolate the effects of BF-DETR and DAPS: the baseline, DAPS only, BF-DETR only, and both components. This design compared the independent and combined contributions of detection and appearance representation. Table 9 reports the results.

The DAPS inference feature extractor contains 26.81 M parameters, excluding unused classification heads. Its computational complexity was estimated using THOP as 8.33 GFLOPs per fish crop, counting one MAC as two FLOPs. The mean feature extraction time was 5.92 ms per fish at batch size 1. At batch size 16, extraction took 24.94 ms per batch, equivalent to 1.56 ms per fish. The latter represents amortized processing time rather than single-fish latency. Extraction timing includes preprocessing, inference, and data transfers.

In Table 9, “Time” denotes the mean processing time per frame for the complete pipeline. The BF-DETR–DAPS–BoT-SORT framework achieved 46.00 ms per frame, corresponding to 21.74 FPS. Pipeline timing includes image-frame reading and JPEG decoding, detection, online ReID feature extraction, and tracking, excluding initialization, visualization, and result writing. Configurations without DAPS retain the original BoT/FastReID feature extractor, whose runtime is included in the pipeline measurements.

With the original detector unchanged, adding only DAPS increased MOTA from 91.6% to 91.7%, HOTA from 66.9% to 69.5%, and IDF1 from 79.3% to 83.4%. Identity switches decreased from 36 to 30. DAPS produced only a small gain in overall tracking accuracy but clearer improvements in association and identity consistency. This pattern is expected because DAPS primarily improves appearance representations used for identity-aware data association.

Adding only BF-DETR increased MOTA, HOTA, and IDF1 to 93.0%, 74.4%, and 89.1%, respectively, while reducing identity switches from 36 to 18. The larger gain from the detector highlights the importance of reliable observations for track initialization and subsequent association. By improving detection accuracy, reducing misses, and localizing dense targets more accurately, BF-DETR reduced fragmentation and erroneous associations caused by detection errors.

Combining BF-DETR and DAPS produced the best performance, with MOTA, HOTA, and IDF1 values of 93.1%, 75.8%, and 91.2% and 13 identity switches. Relative to BF-DETR alone, DAPS improved HOTA and IDF1 by 1.4 and 2.1 percentage points and reduced identity switches by five. DAPS therefore further improved trajectory continuity and identity consistency after detection quality had increased. The modest MOTA gain is expected because MOTA primarily reflects overall detection and tracking accuracy, whereas HOTA and IDF1 more directly capture association and identity quality.

The four ablations show that BF-DETR and DAPS improve fish MOT through complementary mechanisms. BF-DETR reduces the effect of detection errors on track formation, while DAPS distinguishes visually similar individuals. Their combination produced the strongest overall tracking performance, supporting the effectiveness of the BF-DETR-DAPS-BoT-SORT framework under the evaluated conditions.

#### 3.5.4. Visualization of Tracking Improvements Under Challenging Scenarios

**Figure 12:**
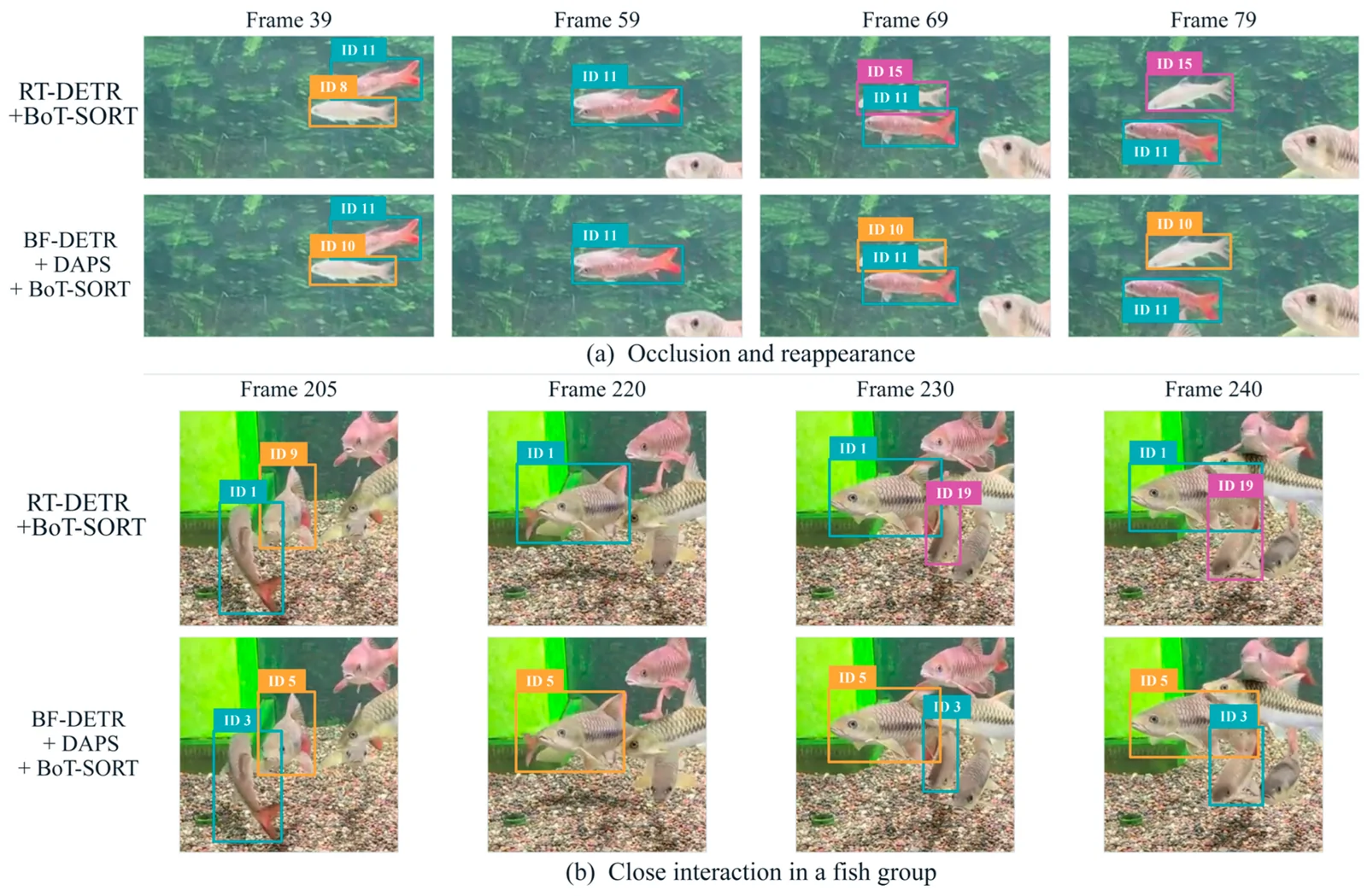
Qualitative comparison of tracking performance between RT-DETR + BoT-SORT and BF-DETR + DAPS + BoT-SORT under challenging scenarios.

To further assess tracking stability in complex underwater environments, we examined two representative challenges: occlusion and reappearance, and close interaction within a fish group. Figure 12 compares the baseline RT-DETR + BoT-SORT framework with the proposed BF-DETR + DAPS + BoT-SORT method.

In the occlusion-and-reappearance scenario (Figure 12a), a fish was obscured by another individual and later reappeared. RT-DETR + BoT-SORT redetected the target in some frames but changed its identity from ID 11 to ID 15 after reappearance, interrupting trajectory continuity. In contrast, BF-DETR supplied stable localization and DAPS provided discriminative appearance features. The proposed method consequently recovered the original identity after occlusion and maintained a more continuous trajectory.

In the close-interaction scenario (Figure 12b), appearance similarity and mutual occlusion increased association errors as several fish approached one another. RT-DETR + BoT-SORT changed a target from ID 9 to ID 19, causing identity drift. DAPS improved appearance discrimination between neighbors, allowing the proposed method to preserve the correct associations during close interaction.

Overall, the proposed method maintained identity more consistently during occlusion, reappearance, and dense interaction. The qualitative evidence supports complementary contributions from BF-DETR detection and DAPS appearance representation. It is also consistent with the higher HOTA and IDF1 and fewer identity switches observed quantitatively.

## 4. Discussion

MOT has become an important tool for intelligent aquaculture monitoring and individual fish behavior analysis [1,4,6]. Unlike manual observation, vision-based tracking can acquire continuous trajectories without disrupting natural activity. These trajectories support behavioral analysis, growth assessment, and farm management [1,2,38]. Nevertheless, underwater environments remain challenging. Light attenuation, reflections, and background changes weaken fish features, while high appearance similarity, frequent occlusion, and crossing cause missed detections, identity switches, and trajectory fragmentation.

Recent studies have advanced fish MOT through improved detectors, spatiotemporal modeling, and association strategies [3,4,6,13]. However, existing approaches often emphasize localization or motion, leaving identity consistency in dense groups insufficiently addressed. Similar body shapes and textures make motion-only association vulnerable to occlusion, pose changes, and crossing [10,13]. Reliable long-term tracking therefore requires discriminative individual representations together with accurate detection.

We developed BF-DETR-DAPS-BoT-SORT to integrate detection enhancement, individual re-identification, and trajectory association. BF-DETR improves localization reliability in complex underwater scenes, while DAPS strengthens appearance discrimination between similar individuals. The experiments indicate complementary effects. Improving detection alone did not fully resolve identity preservation, whereas discriminative appearance features increased trajectory continuity in challenging scenes.

The effectiveness of the proposed framework is primarily reflected in the coordinated improvement of detection and identity association. Detection errors in dense groups disrupt track initialization and matching, while weak identity cues promote identity switches after targets cross. BF-DETR stabilizes target observations by strengthening fish structure and boundaries. DAPS supplies additional identity constraints to BoT-SORT through individual appearance information. Their combination maintained identity more consistently under occlusion, close interaction, and pose variation in the evaluated tank scenes.

Fish-specific ReID approaches share the goal of distinguishing individuals through subtle appearance differences, but employ different representation strategies. Fan et al. [10] introduced FSNet to integrate multiview information. Haurum et al. [39] combined color and texture descriptors with metric learning for zebrafish ReID, while Zhao et al. [40] integrated global detail enhancement with keypoint-region features. DAPS also combines global and local appearance information, but constructs its local representations through pooling along two fixed image-coordinate axes without explicit keypoint localization. During training, forward and reversed correspondence are compared only for the six longitudinal tokens to accommodate opposite head–tail ordering. This distinguishes its local correspondence modeling from the multiview integration, descriptor-based metric learning, and keypoint-guided feature extraction used in these representative approaches.

Continuous identity-preserving trajectories provide a basis for individual behavioral analysis. Identity switches can introduce artificial jumps into movement records, while trajectory fragmentation interrupts the estimation of activity duration. With appropriate temporal and spatial calibration, trajectories could support measurements of projected swimming speed, turning frequency, and movement-bout duration. For schooling behavior, consistent identities could help characterize changes in projected nearest-neighbor relationships and movement alignment over time. In feeding trials with known feed-delivery times and a defined feeding region, potential response metrics include latency to enter the feeding region, residence time, and changes in movement before and after feeding.

These applications require validation beyond tracking accuracy. The present single-view trajectories describe projected movement and do not directly measure swimming energy expenditure, feed intake, or welfare status. Interpreting movement in energetic terms would require a validated relationship with physiological measurements, while feeding-response metrics would require synchronized feeding observations. Future work will examine whether improved trajectory continuity produces more reliable behavioral measurements under these conditions.

This study has several limitations. The data were collected primarily in laboratory tanks and included scenes involving occlusion, dense groups, and reflection interference. Differences in water quality, illumination, and species diversity may limit generalization to production environments. The current ReID evaluation reports aggregate retrieval performance and does not separately quantify robustness under direction changes or occlusion. The contribution of each component under these conditions therefore remains to be established. Future work will define explicit condition criteria, construct annotated evaluation subsets, and compare the model variants under a consistent protocol. In addition, combining detection and ReID networks increases computational cost. Future work should evaluate additional species and production environments and investigate lightweight architectures and multisource information for practical deployment.

## 5. Conclusions

We proposed BF-DETR-DAPS-BoT-SORT for fish multi-object tracking in complex underwater environments. The framework addresses unstable detection and difficulty in maintaining identity consistency by jointly strengthening target localization and individual representation. It consequently improves tracking stability in dense multi-fish scenes.

At the detection stage, BF-DETR builds on RT-DETR-R18 and uses FMEM and BCSM to strengthen fish morphological representations and boundary discrimination. This design improves robustness to degraded visual features and high-density scenes. BF-DETR achieved 95.17% Precision, 93.40% Recall, 96.63% mAP@0.5, and 77.53% mAP@0.5:0.95, providing reliable observations for subsequent tracking.

At the tracking stage, DAPS learns discriminative appearance features and supplies them to BoT-SORT for data association. BF-DETR-DAPS-BoT-SORT achieved a HOTA of 75.8%, MOTA of 93.1%, and IDF1 of 91.2%, with 13 identity switches. These results indicate that enhanced appearance representations improved trajectory continuity and identity consistency in the evaluated complex multi-fish underwater scenes.

Overall, BF-DETR-DAPS-BoT-SORT coordinates fish detection and identity representation to support stable multi-object tracking under occlusion, high-density conditions, and appearance similarity. Within the evaluated conditions, the framework supports subsequent fish behavior analysis, growth monitoring, and intelligent aquaculture management.

## Figures and Tables

**Table 1:** Statistics of the fish detection dataset.

Dataset	Recordings	Original Images	Augmented Images	Total Images	Fish Instances
Train	12	1372	1670	3042	39,918
Validation	4	171	0	171	2256
Test	4	172	0	172	2274
Total	20	1715	1670	3385	44,448

**Table 2:** Statistics of the fish multi-object tracking dataset.

Split	Recordings	Frames	Identities	Bounding Boxes
Training	4	4567	42	42,562
Validation	4	1516	41	14,556
Testing	4	1343	42	13,906
Total	12	7426	125	71,024

**Table 3:** Statistics of the fish re-identification dataset.

Split	Identities	Images
Train	29	6253
Query	13	378
Gallery	13	1138
Total	42	7769

**Table 4:** Performance comparison of different object detection models.

Model	Precision	Recall	F1	mAP@0.5	mAP@0.5:0.95	Params
Faster R-CNN	70.81	86.50	77.87	87.23	49.92	41.48
YOLOv9m	92.63	89.10	90.83	94.64	71.24	20.04
YOLOv10m	92.39	88.16	90.23	95.05	72.76	15.33
YOLOv11m	93.98	89.01	91.43	95.54	73.50	20.06
YOLOv12m	93.60	89.23	91.36	95.42	73.35	19.58
D-FINE-M	91.25	91.20	91.22	95.00	73.20	19.00
RT-DETR-L	94.66	91.13	92.86	95.69	75.13	32.06
RT-DETR-R34	94.30	92.00	93.13	95.88	74.91	31.16
RT-DETR-R18	93.69	90.76	92.20	94.90	72.90	19.90
BF-DETR	95.17	93.40	94.28	96.63	77.53	21.15

**Table 5:** Ablation results of FMEM and BCSM.

Use RT-DETR-R18	FMEM	BCSM	Precision	Recall	F1	mAP@0.5	mAP@0.5:0.95	Params
✓	×	×	93.69	90.76	92.20	94.90	72.90	19.90
✓	✓	×	95.21	91.80	93.47	95.67	75.16	19.97
✓	×	✓	95.63	91.50	93.52	96.15	76.16	21.06
✓	✓	✓	95.17	93.40	94.28	96.63	77.53	21.15

**Table 6:** Performance comparison of different ReID methods.

Model	mAP	Rank-1	Rank-5	Rank-10
ResNet-50	65.83	75.39	87.03	92.85
PCB	67.12	79.62	94.70	97.88
AlignedReIDNet	69.80	83.60	91.00	94.40
MGN	70.24	81.22	93.12	95.50
DAPS	71.76	85.45	91.80	94.97

**Table 7:** Ablation study of individual components in DAPS.

UTDE	Dual-Axis Representation	Reverse Part Matching	mAP	Rank-1
×	×	×	65.83	75.39
×	✓	✓	69.18	82.01
✓	×	✓	69.87	82.80
✓	✓	×	70.02	82.54
✓	✓	✓	71.76	85.45

**Table 8:** Performance comparison of different multi-object tracking algorithms.

Model	MOTA	HOTA	IDF1	IDSW
ByteTrack	90.8	67.4	84.6	24
OC-SORT	92.5	71.3	83.2	20
StrongSORT	91.2	67.0	77.5	84
BoT-SORT	93.0	74.4	89.1	18
DeepSORT	89.2	59.8	72.2	106
BoostTrack	92.6	71.2	84.1	26
Ours	93.1	75.8	91.2	13

**Table 9:** Ablation study of BF-DETR and DAPS with tracking performance and runtime. ↑ indicates that higher values are better, and ↓ indicates that lower values are better.

BoT-SORT-Based Setting	BF-DETR	DAPS	MOTA ↑	HOTA ↑	IDF1 ↑	IDSW ↓	Time ↓	FPS ↑
✓	×	×	91.6	66.9	79.3	36	50.69	19.73
✓	×	✓	91.7	69.5	83.4	30	45.98	21.75
✓	✓	×	93.0	74.4	89.1	18	51.46	19.43
✓	✓	✓	93.1	75.8	91.2	13	46.00	21.74

## Data Availability

The data that support the findings of this study are available from the corresponding author upon reasonable request.
